# Efficacy and Safety of Argon Plasma Coagulation for Hemorrhagic Chronic Radiation Proctopathy: A Systematic Review

**DOI:** 10.1155/2018/3087603

**Published:** 2018-02-25

**Authors:** Yanan Peng, Haizhou Wang, Juerong Feng, Shilin Fang, Meng Zhang, Fan Wang, Ying Chang, Xianyan Shi, Qiu Zhao, Jing Liu

**Affiliations:** ^1^Department of Gastroenterology, Zhongnan Hospital of Wuhan University, Wuhan, China; ^2^Hubei Clinical Center and Key Lab of Intestinal and Colorectal Diseases, Wuhan, China

## Abstract

Hemorrhagic chronic radiation proctopathy (CRP) is a common complication after pelvic radiotherapy in patients with prostate or gynecological cancers. This systematic review was conducted to evaluate the efficacy and safety of argon plasma coagulation (APC) in treating hemorrhagic CRP. The databases of PubMed, Embase, and Cochrane Library were searched for related studies from inception to July 2017. Finally, 33 studies were identified with a total of 821 hemorrhagic CRP patients. After APC treatment, hemoglobin levels increased from 7.7–13.4 g/L to 11–14 g/L (including 15 studies). All (*n* = 33) studies reported an effective rate in rectal bleeding, among which five studies had a rate of 100%. Short-term complications were reported in 31 studies, while long-term complications in 33 studies and no complication in 11 studies. As for the severe complications, perforation was reported by 2 out of 33 studies, and the incidences were 3.3% (1/30) and 3.7% (1/27), respectively. As for APC setting, argon gas flow rate (median 1.5 L/min) and electric power (median 50 W) had no significant influence on complications and hemostasis. In conclusion, current literature indicated that APC therapy was an effective and safe strategy for hemorrhagic CRP, and large-scale prospective studies are needed to warrant our study.

## 1. Introduction

Hemorrhagic chronic radiation proctopathy (CRP) is a common complication after pelvic radiotherapy. It had an incidence of 5% to 15% in the patients of pelvic cancers within 6 months after radiotherapy [1]. Multiple factors were involved in the pathogenesis of hemorrhagic CRP, like mucosal damage, microvascular injury with tissue ischemia, and telangiectasias [2]. Oral or enema treatment with salicylates, corticosteroids, and sucralfate usually had a limited benefit. Moreover, surgery was not recommended for a high incidence of morbidity [3]. However, hyperbaric oxygen therapy (HOT) and formalin application were reported to be effective in radiation proctitis [3–8]. As an easy method in destructing the telangiectasias caused by radiotherapy, argon plasma coagulation (APC) was reported to have a better efficacy than formalin in treating hemorrhagic CRP (79% versus 27%, *P* = 0.017) [4]. Alvaro et al. also found that APC therapy had a significantly better response than hyperbaric oxygen therapy in reducing blood transfusion and tissue toxicity, although both treatments showed no significant difference in resolving rectal bleeding. In spite of this, several studies still reported a high incidence of up to 20% in APC-associated complications, such as ulcers, perforations, strictures, and fistulas [5–10]. Furthermore, Sato et al. found that appropriate APC settings (e.g., electric power, application time, and argon flow rate) could reduce the damage to deeper tissues, thus decreasing the incidence of complications [11]. However, its effectiveness and safety were not clearly assessed in previous studies. Therefore, we conducted a systematic review to evaluate the efficacy and safety of APC in treating hemorrhagic CRP.

## 2. Methods

### 2.1. Medical Literature Search

The databases of PubMed, Embase, and Cochrane Library were searched for relevant studies from inception to July 2017, using the key words including “radiation proctopathy” [MeSH Terms] OR (“radiation” [All Fields] AND “proctopathy” [All Fields]) OR “radiation proctopathy” [All Fields] OR (“radiation proctitis” [MeSH Terms]) OR (“radiation” [All Fields] AND “proctitis”[All Fields]) OR (“radiation proctitis” [All Fields]) AND (“Argon plasma coagulation therapy” [MeSH Terms] OR (“Argon plasma coagulation” [All Fields] AND “therapy” [All Fields]) OR “Argon plasma coagulation therapy” [All Fields]) OR (“APC therapy”[MeSH Terms]) OR (“APC therapy” [All Fields]). There was no language restriction for literature search.

### 2.2. Selection Criteria and Exclusion Criteria

All the studies were reviewed independently by two investigators. Studies were included if fulfilling the following criteria: (i) including patients with pelvic cancers and subsequent radiotherapy, (ii) diagnosed as hemorrhagic CRP, and (iii) evaluated the efficacy and safety of APC. The exclusion criteria were as follows: reviews, case reports, animal studies, and studies without full text or sufficient data.

### 2.3. Data Extraction and Quality Assessment

The following information was extracted from each included study: author, year of publication, area, study design, number of cases, age, gender, cancer type, follow-up time, argon gas flow rate and electric power of APC, number of rectal bleeding cessation (NRBC), hemoglobin (LHb) levels, and short-term and long-term complications. The quality of included studies were assessed using an adjusted version of the Newcastle-Ottawa Scale, which contained a cohort selection on APC administration and its representativeness, ascertainment of APC exposure, and evidence that there was no prior exposure to APC, outcome, longevity follow-up (at least 6 weeks), and bias due to dropout or incomplete follow-up [12].

### 2.4. Statistical Analysis

The efficacy of APC therapy was evaluated according to the degree of improvement in rectal bleeding and hemoglobin, while the safety was assessed based on the incidence of complications, which were categorized as short term (e.g., urgency, diarrhea, incontinence, fever, anal or abdominal pain, and perforation) and long term (e.g., stenosis/strictures, rectal ulcers, recurrence of rectal bleeding, and fistula). As for APC settings, electric power, argon gas flow, and coagulation time were included analysis. Studies with a score of ≥5 were considered as high quality, with 3-4 for moderate quality. Discrepancies of assessments were resolved through discussion.

## 3. Results

### 3.1. Study Characteristics

**Figure 1:**
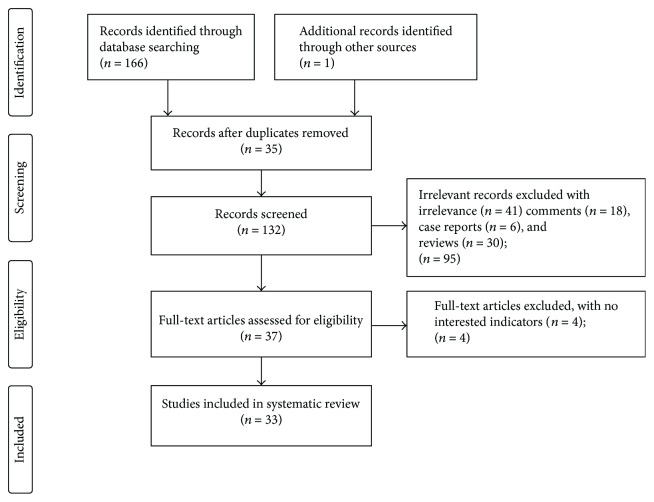
Flow diagram of the identification of the studies for inclusion in the systematic review.

Of the 167 papers identified at the initial search, after removing the duplicates and exclusion of irrelevant studies, comments, case reports, and reviews, 37 studies were selected for the review of the full text. Another 4 studies were excluded due to the absence of certain interested outcomes including rectal bleeding cessation, the improvement of hemoglobin, and short-term and long-term complications, and 33 studies were included in the final review (Figure 1). All included studies including prospective (*n* = 21) and retrospective (*n* = 8) uncontrolled cohort trials, and 4 nonrandomized control trials [4, 13–15], were conducted at a single academic center. The average time of follow-up was 28 months (1 month to 170 months) (Table 1). All patients with chronic radiation proctitis were characterized by rectal bleeding, which was referred to as hemorrhagic CRP.

### 3.2. Efficacy of APC

The improvements in rectal bleeding and hemoglobin were regarded as efficacy of APC treatment, which were observed in 821 and 383 patients, respectively. 15 studies reported the improvement of hemoglobin after APC administration.  Table 2 demonstrates that after APC treatment, the mean of hemoglobin levels was improved from 7.7–13.4 g/L to 11–14 g/L. 33 studies documented rectal bleeding cessation as evidenced in Table 2, 5 of which had a 100% hemostasis rate [5, 7–9, 16]. Furthermore, in the largest study, Sato et al. [11] reported that during a mean follow-up of 34.6 months, 4 patients (6.3%) had minor recurrent rectal bleeding and 60 (93.8%) remained in remission. (*P* = 0.002) (Table 2).

### 3.3. Safety of APC

APC-associated short-term and long-term complications were shown in Table 3. Short-term complications were reported in 31 studies, while long-term complications in 33 studies and no complication in 11 studies. The incidence of complications was 0–63.6%. Especially in a study from Venkatesh and Ramanujam, USA, there was no complication among 40 patients after APC therapy [17], because the team had experience. The commonest procedure-related short-term complications reported were anal or rectal pain with or without tenesmus, which was most likely to occur following treatment near the dentate line. Abdominal bloating, vomiting, adynamic ileus, vagal symptoms, cramping, incontinence, fever, and colonic explosion were also reported. Necrosis of the lower part of the rectum was reported in one study [10]. The severe complication of perforation was reported in 2 series [10, 18], the incidence of which was 3.7% (1/27) and 3.3% (1/30), respectively. Ben-Soussan et al. reported that one patient had, during APC, colonic explosion which immediately led to a perforation [18]. The patient needed surgical treatment and made a complete recovery in 2 weeks. At the same time, Ben mentioned that the incidence of bowel explosion was higher after local preparation in comparison with oral reparation (*P* < 0.05). Enema preparation with persistent solid stool above the coagulated lesions contributed to the main risk of perforation. Canard et al. also reported 1 perforation [10]. But we could not obtain the detailed information about the treatment of perforation and prognosis of the patient from their study. Grund et al. reported 1600 applications of APC for a variety of indications in the upper and lower gastrointestinal tract and reported a perforation rate of 0.31% [19].

Rectal ulcers, stenosis, and recurrence of rectal bleeding were common long-term complications following APC treatment. Chruscielewska-Kiliszek et al. reported rectal ulcers in 35 (56.5%) of 62 patients [20], an incidence that was relatively high in comparison with the reported overall rate of about 3.3% (1/30) [14]—21.4% [13] (Table 3) in other series. Rectal ulcers developing during APC could be considered a consequence of thermal injury to already damaged and vascularly compromised tissue that was thus more fragile and had poorer healing [21]. The fact that rectal ulcers were not clinically troublesome meant they should not be considered an absolute contraindication to APC nor do they necessarily require any additional endoscopic follow-up [22]. The occurrence of stenosis compared with rectal ulcer was less common. The incidence of rectal stenosis varied among different studies, many studies describing no occurrence of rectal stricture while few studies reporting such complication in 2% (1/49) [23]—13.3% (2/15) [7]. However, given the fact that most of the rectal strictures were asymptomatic, their true incidence was difficult to estimate and theoretically would be higher than reported by several studies. The recurrence rate of rectal bleeding ranged from 2.1% (1/48) [24] to 6.3% (1/16) [6]. Patients on anticoagulants or aspirin were more likely to relapse. Kaassis et al. reported that patients who were receiving anticoagulation therapy required more APC sessions but could also achieve an equivalent therapeutic efficacy as those who were not on anticoagulation [6].

### 3.4. Arguments of APC Setting

By reviewing relative literatures, we realized that different studies had different optional APC settings. This systematic review showed arguments of APC settings in 32 trials (Table 1). The electric power setting ranged from 25 to 80 W (median 50 W), with an argon flow rate from 0.6 to 3.0 L/min (median 1.5 L/min). Of 10 trials that reported the records of coagulation time, only one trial briefly provided the specific number of coagulation time. The range of coagulation time was from 0.5 s to 3 s. Among included studies that observed incidence of complications, regardless of the range of electric power setting (30–50 W versus 50–80 W), the corresponding rate of complications had no difference (0–58.1% versus 0–63.6%). Even with the same power (60 W) and the flow rate > 1.5 L/min, 4 series, respectively, reported complications in 0%, 35.7%, 0%, and 13.3% [5, 7, 13, 25]. In addition, there were no uniform settings of APC in 5 series which reported 100% hemostasis rate. Therefore, APC settings appeared to be uncorrelated with the incidence of complications in our study.

### 3.5. Quality Assessment

The quality assessment of studies using NOS is shown in Table 4. The qualities of studies were considered high for 31 studies and moderate for 2 studies.

## 4. Discussion

Chronic radiation proctopathy had an incidence of 5%–15% in patients with pelvic radiotherapy, and rectal bleeding was the most common complication. Radiation injury to the rectal wall became apparent like obliterative endarteritis with secondary tissue ischemic and development of neovascular mucosal lesions. These ones could bleed in a delayed fashion and different amounts: from little sporadic spotting which leads, sometimes to chronic anemia state, to episodes of severe rectal bleeding [26].

APC is a nontouch electrocoagulation technique in which high-frequency alternating current can be delivered to the target lesion by ionized gas. The limited depth of coagulation (0.5–3 mm) [27] explained the low risk of perforation, stenosis, and fistulization. Unlike traditional bipolar devices, APC could be applied axially and radially, allowing tangential coagulation of lesions around rectal bends without significant reduction in effectiveness. Moreover, the APC generator is mobile and can be used quickly at any place or time [28]. Thus, APC is a well-established treatment for various conditions, such as oozing hemorrhage from angiodysplastic lesions or polypectomy sites.

Our study firstly confirmed that APC was an effective and safe therapy in endoscopically treating hemorrhagic CRP. APC therapy had a high success rate of hemostasis and low incidence of complications, which could help improve the hemoglobin levels. Furthermore, APC therapy could be performed at the outpatient clinics because no sedation or analgesia was required during the procedure [29]. Sedation with midazolam, fentanyl, or propofol was administrated to minimize the discomfort caused by the lesions near dentate line or gaseous distension of the rectum [7, 9, 30].

Several studies reported that all patients had a decrease in transfusional requirements and an improvement in anemia [6, 7, 10, 16, 25]. The median lowest hemoglobin levels were 9.6 g/dL (range 5.1–14.1) before APC and the median improvement in hemoglobin levels after treatment was 2.05 g/dL (range 0.5–5.1) in a study by Hortelano et al. [14]. As for safety, more than ten studies reported no complication during the follow-up [5, 15–17, 25, 31–36]. However, several studies reported a low incidence of complications, including rectal or anal pain, recurrence of bleeding, rectal ulceration, and anal or rectal strictures. Rectal pain usually occurred near the dentate line after APC treatment [9]. It could be resolved spontaneously within a few days or with standard analgesics [9, 10, 29]. Recurrence of rectal bleeding was most likely to happen when patients were on anticoagulants or aspirin, and it also could be successfully treated after additional APC treatment. Although rectal ulcer and stenosis were common long-term complications, they were generally asymptomatic and did not require any additional endoscopic follow-up. In this systematic review, necrosis of the lower part of the rectum was reported in only one study [10]. 2 studies [10, 18] reported perforation following APC with low incidence. Ben et al. reported that one patient sustained a colonic explosion complicated by perforation that needed urgent surgery. The pathophysiology of the colon explosion remained unclear, but an accumulation of colonic gas (hydrogen and methane) at potentially explosive concentrations [37–39] could be the cause, especially the presence of stools above the lesions.

The optimal number of treatment sessions was still unknown. APC was traditionally not applied in one treatment session, particularly in patients with severe diseases. For therapeutic success, the median number of sessions per patient ranged from 1 to 3.7. Swan et al. [23] documented that there was a significant improvement in rectal bleeding among 68% of patients after the first session and 96% after two sessions. Karamanolis et al. [40] reported that APC with 2 sessions could resolve rectal bleeding completely in 89.3% (50/56) patients. According to this systematic review, the mean value of APC sessions was 1.9.

As for APC setting, there was still no consensus for the optimal APC settings (power and gas flow rate) for successful and safe coagulation. Sato et al. [11] reported that the ideal coagulation time was 2 seconds from an ex vivo experiment on swine rectal mucosa. Weiner et al. [41] also found the impact of coagulation time on the depth and diameter of the coagulation zone and reported that as coagulation time and electric power increase, at larger electric power (>75 W) and/or longer coagulation time (>3 s), the formation of craters and artificial clefts in submucosa and perforation increase. Because the data of coagulation time were not available in included studies, we could not conduct an analysis which could explain the effect of coagulation time on the depth. To sum up, coagulation time ranged from 0.5 s to 3 s in our study. Canard et al. reported that lower power settings were subscribed for a lower complication rate and decreased number of treatment sessions required for complete coagulation, with almost all complications occurring at power settings above 45 W [10]. We generally thought that lower power settings caused less injury, while in this systematic review, APC settings seemed to have no impact on complications.

We intended to further study the difference between the ICC system (e.g., first-generation APC) and the second-generation APC. Unfortunately, all of the trials included used the ICC system with parameter settings that were distinctly different from the second-generation APC. Compared with the ICC system, the second-generation APC offered a broader bandwidth of parameters including different APC modes and a range of power settings from 1 to 120 W [42]. Different APC modes appeared to be safe and effective in a variety of gastrointestinal conditions [43, 44].

Other than APC, salicylates, corticosteroids, sucralfate, and short-chain fatty acid enemas have been used with limited success, but hyperbaric oxygen therapy (HOT) and formalin application were shown to be effective in radiation proctitis [4, 5, 45–48].

The mechanism of HOT in treating radiation tissue injury was the induction of neovascularization which could reverse tissue hypoxia. Macrophages responding to the oxygen gradient between the damaged hypoxic cells and the surrounding normal tissue mediated the stimulus for angiogenesis [49]. Tahir et al. [50] reported a 95% efficacy of HOT for hemorrhagic CRP, where around half of the cases had a durable major response. Some patients even experienced symptom relief lasting as long as seven years. However, before consenting to HOT, patients should consider these factors: (1) Pressure inside the hyperbaric chamber can damage the middle and inner ear, nasal sinuses, lungs, and teeth in both adults and children. (2) Some people experienced claustrophobia inside the chamber. (3) The therapy might affect your eyes, for example, by promoting nearsightedness or cataract growth. (4) Diabetics should have their levels checked before and after treatment, because hyperbaric oxygen therapy affects blood sugar levels. (5) The cost of HOT was high enough, and it was not widely applicable. There was no uniformity in the methods of HOT. Although it could be perceived from the studies that HOT was useful in refractory radiation proctitis, there was marked variation between the studies [48, 50–53]. The reported number of HOT sessions for a successful treatment ranges from 12 to 90. Recently, a double-blind, sham-controlled, phase-3 randomized trial conducted by Glover et al. [54] reported that there was no significant difference between APC and HOT in rectal bleeding. Álvaro-Villegas et al. reported that APC and HOT were similar in treating rectal bleeding, while response rate was higher and faster in the APC group [13].

Formalin was a mixture of methanol and formaldehyde which covalently binded to proteins and causes cell necrosis. It acted as a hemostatic agent causing chemical cauterization to control bleeding from telangiectatic mucosal and submucosal vessels. In 1986, Rubinstein et al. were the first to use formalin for a hemorrhagic CRP patient to get a good response [55]. Most used 4% dilute formalin applied to the rectum mucosa either by direct application of formalin-soaked gauze or by “instilling” the solution in single or multiple aliquots down the operating channel of a colonoscope. Guo et al. in their randomized trial which randomly divided 122 patients into 4% or 10% formalin application showed that 10% formalin was associated with complications and 4% formalin should be the choice for treating hemorrhagic CRP [56]. Alfadhli et al. [4] compared the efficacy of formalin instillation therapy with APC therapy and found that APC showed a better efficacy (78.5% versus 27%). Nevertheless, the study of Yeoh et al. [57] found that there was no statistical difference between the APC group and the formalin group (94% versus 100%, respectively). It was worth noting that in formalin therapy, 18% of the patients underwent intestinal stricture and 21% with fecal incontinence [58]. Therefore, formalin might be proper for patients with proctitis and refractoriness to other endoscopy therapies, like argon plasma coagulation, rather than an upfront approach.

Any randomized control trials on APC in treating hemorrhagic CRP could not be available, which had the underlying methodological limitations in this systematic review. In addition, individual patient data (IPD) from eligible trials on APC were not available because it might not be possible to contact the original trial authors or authors might not be willing to share the raw data. Furthermore, we included several trials published early.

In conclusion, APC is a safe and effective method for treating hemorrhagic CRP. Further evidence from randomized controlled trials and comparative studies is required to confirm the role of APC and APC settings, and APC should be considered as a first-line therapy for hemorrhagic CRP.

## Figures and Tables

**Table 1:** Characteristics of studies included in the systematic review.

First author (year, area)	Study design	Age	Cancer types	No. of patients	Electric power (W: watt)	Argon gas flow rate (L/min)	Rate of complication	Frequencies of session	Rate of hemostasis	Follow-up (m: month)	Loss-up
Higuera (2004, Spain)	Retrospective	67.8 (58–76)	Cervical (1), endometrial (6), >and prostate (3) cancer	10	60	1.5–2	0.0%	1.9 (1–4)	78.6	31.8 m (10–45 m)	0
Alfadhli (2008, Kuwait)	Prospective	74.7	na	14	45–50	1.2–2	14.3%	1.78	85.7	3 m	0
Alvaro (2011, Mexico)	Prospective	59.3 ± 12	na	14	60	1.6	35.7%	3 ± 1	92.6	3 m	0
Ben (2004, France)	Prospective	73.1 (53–86)	Prostate (19), anal (4), uterine (3), and rectal (1) cancer	27	40–50	0.8–1	18.5%	2.66 (1–7)	92.9	13.6 m (3–31 m)	0
Canard (2003, France)	Prospective	70.7 (58–85)	Prostate (23), uterine (4), cervical (1), and squamous-cell anus (1) cancer and uterine (1) sarcoma	30	42 (30–80)	1.5 (0.8–1.2)	26.7%	69, 2.3 (1–5)	95.2	1–35 m	2
Chruscielewska (2013, Poland)	Prospective	65.5 ± 10.9	Cervical (16), endometrial (17), prostate (28), and rectal (1) cancer	62	25–40	1.5–2/1–1.5	58.1%	2 (1–3)	91.7	52 weeks	0
Chutkan (1997, USA)	Prospective	72 (64–85)	Prostate (10) and uterine (2) cancer	12	na	na	0.0%	1 (1-2)	97.9	6.6 m (3–13 m)	0
Dees (2006, Netherlands)	Prospective	73.6 (59–89)	Prostate (45), urinary bladder (4), and uterine cervix (1) cancer	50	50	2	2.0%	3 (1–6)	100	na	2
Fantin (1999, Switzerland)	Retrospective	na	Prostate (6) and endometrial cancer (1)	7	60	3	0.0%	2 (2–4)	90	24 m (18–24 m)	0
Hortelano (2014, Spain)	Retrospective	70 (56–78)	Prostate cancer	30	50	1.8	6.67%	3	76.7	14.5 m (2–61 m)	0
Kaassis (2000, France)	Retrospective	73.5 (62–80)	Prostate (15) and uterine (1) cancer	16	40	0.6	31.3%	3.7	93.8	10.7 m (8–28 m)	0
Karamanolis (2009, Greece)	Prospective	68.4 (45–86)	Prostate cancer	56	40	2	5.4%	2 (1–8)	89.3	17.9 m (12–33 m)	0
Latorre (2008, Spain)	Prospective	70.9 ± 7.38	na	38	50–60	1.4–1.8	0.0%	3.6 ± 2.7	86.8	28.5 ± 3.9 m	0
Lenz (2011, Brazil)	Prospective	70.4 ± 11.1	Prostate (8), cervical (5), and endometrial (2) cancer	15	40	1	40%	3.7 ± 1.7	93.3	12.5 m (2–30 m)	0
Lpoez (2010, Mexico)	Retrospective	64 (25–80)	Cervicouterine (5), endometrial (2), vaginal (2), prostate (9), and colorectal (1) cancer	19	40–50	1–1.5	5.3%	2 (1–7)	94.7	29 m (1–93 m)	0
Onoyama (2011, Japan)	Prospective	74 ± 5.5	Prostate cancer	24	30–40	1	0.0%	(1–7)	100	23.5 m (1–53 m)	0
Rolachon (2000, France)	Prospective	70.3 ± 10	Prostate (11) and endometrial (1) cancer	12	50	1	25.0%	2.8 ± 0.8	91.7	6 m	0
Rotondano (2003, Italy)	Prospective	69.2 (22–81)	Endometrial (13), cervical (6), and prostate (5) cancer	24	40	0.8–1.2	25.0%	69, 2.5 (1–6)	91.7	41 m (24–60 m)	0
Sait (2013, Turkey)	Retrospective	61	Prostate (6), rectum (2), cervix (12), and endometrium (1) cancer	21	50 (40–60)	1.5 (1.2–2)	23.8%	3 (1–11)	85.7	34.6 m	0
Samy (2012, Egypt)	Prospective	na	na	23	40–50	0.8–1.0	0.0%	na	73.9	37 m (6–84 m)	0
Sarah (2001, France)	Prospective	73 ± 3	Prostate (9), uterine (1), and rectal (1) cancer	11	50	0.8–2	63.6%	3.2 ± 0.4	100	19 ± 2 m	0
Sato (2011, Japan)	Prospective	72 (35–83)	Prostate (46) and cervical (19) cancer	65	40	1.2	18.5%	2 (1–5)	93.8	34.6 m (3.6–121.1 m)	0
Sebastian (2004, Ireland)	Prospective	69 (53–77)	Prostate (23) and bladder (2) cancer	25	30 (25–50)	1.5	0.0%	1 (1–4)	84	14 m	0
Silva (1999, Portugal)	Prospective	65 (42–77)	Cervical (17), endometrial (7), and prostate (4) cancer	28	50	1.5	10.8%	2.9 (1–8)	96.4	10 m (1–15 m)	0
Smith (2001, USA)	Prospective	na	Prostate cancer	7	40–45	1.6	0.0%	1–3	71.4	4–13 m	0
Swan (2010, Australia)	Prospective	72.1 (51–87)	Prostate (45), uterine (2), cervical (2), and vaginal (1) cancer	50	50	1.4–2.0	36.0%	1.36 (1–3)	98	20.6 m (5–48 m)	1
Takemoto (2012, Japan)	Prospective	na	Prostate cancer	12	30–40	1	0.0%	(1–3)	83.3	35 m (12–69 m)	0
Tam (2000, Australia)	Retrospective	na	Prostate (14) and cervical (1) cancer	15	60	2	13.3%	2 (1–4)	100	24 m (8–35 m)	0
Tjandra (2001, Australia)	Prospective	73 (62–78)	Prostate (10) and cervix (2) cancer	12	40	1.5	0.0%	na	50	11 m (4–17 m)	0
Venkatesh (2002, USA)	Prospective	64–83	na	40	40–60	1.5	0.0%	na	97.5	3–30 m	0
Villavicencio (2002, USA)	Prospective	72.6 (58–86)	Prostate (15), endometrial (4), sacral chondroma (1), and cervical (1) cancer	21	45–50	1.2–2	19.0%	1.7 (1–4)	100	10.5 m (1–29 m)	0
Yeoh (2013, Australia)	Prospective	73 (49–87)	Prostate cancer	17	60–80	2	0.0%	2	94.1	110 m (29–170 m)	0
Zinicola (2003, Italy)	Retrospective	68 (30–80)	Prostate (8), cervical (4), and bladder (2) cancer	14	65	2	7.1%	2.0 (1–4)	83.3	19 m (5–41 m)	2

**Table 2:** Study characteristics of selected studies on the levels of hemoglobin and rectal bleeding before and after APC administration.

Author	Year	Type of study	Proctitis	Total number of bleeding	Number of rebleeding	Rate of hemostasis (%)	Mean level of hemoglobin before APC (g/dL) ± SD	Mean level of hemoglobin after APC (g/dL) ± SD
Alfadhli	2008	Prospective	Prospective	14	3	78.6	na	na
Álvaro-Villegas	2011	Prospective	Chronic radiation proctitis	14	2	85.7	9.9 ± 2.3	11.3 ± 2
Ben	2004	Prospective	Hemorrhagic radiation proctitis	27	2	92.6	na	na
Canard	2003	Prospective	Radiation proctitis	28	2	92.9	na	na
Chruscielewska	2013	Prospective	Chronic radiation proctitis	62	3	95.2	13.07 ± 1.73	13.96 ± 1.44
Chutkan	1997	Prospective	Proctitis	12	1	91.7	na	na
Dees	2006	Prospective	Chronic radiation proctitis	48	1	97.9	na	na
Fantin	1999	Retrospective	Proctitis	7	0	100.0	na	na
de la Serna Higuera	2004	Retrospective	Hemorrhagic radiation proctopathy	10	1	90.0	na	na
Hortelano	2013	Prospective	Chronic radiation proctitis	30	7	76.7	9.6 (5.1–14.1)	11.65 (10.2–14.6)
Kaassis	2000	Retrospective	Proctitis	16	1	93.8	na	na
Karamanolis	2009	Prospective	Radiation proctitis	56	6	89.3	na	na
Latorre	2008	Prospective	Chronic radiation proctopathy	38	5	86.8	11.3 ± 3.05	14.014 ± 1.29
Lenz	2011	Prospective	Chronic radiation coloproctopathy	15	1	93.3	11.7 ± 2.7	13.0 ± 0.9
Lpoez	2010	Retrospective	Radiation proctopathy	19	1	94.7	11.8 (7.3–16.5)	12.9 (7.5–16.5)
Onoyama	2011	Prospective	Chronic hemorrhagic radiation proctitis	24	0	100.0	10 ± 2.2	12.3 ± 1.5
Rolachon A	2000	Prospective	Proctitis and proctosigmoiditis	12	1	91.7	7.9 ± 2.1	11 ± 1.4
Rotondano	2003	Prospective	Chronic radiation proctopathy	24	2	91.7	9.2 ± 2.4	13.6 ± 1.1
Sait Dag	2013	Retrospective	Radiation proctitis	21	3	85.7	na	na
Samy	2012	Prospective	Chronic proctitis	23	6	73.9	na	na
Sarah	2001	Prospective	Proctitis	11	0	100.0	7.7 ± 2.8	11.5 ± 2.6
Sato	2011	Prospective	Hemorrhagic radiation proctopathy	65	4	93.8	11.1 (5.8–13.3)	13.7 (12–15.2)
Sebastian	2004	Prospective	Radiation proctopathy	25	4	84.0	10.05 ± 2.21	12.44 ± 1.09
Silva	1999	Prospective	Proctosigmoiditis	28	1	96.4	na	na
Smith	2001	Prospective	Proctitis	7	2	71.4	na	na
Swan	2010	Prospective	Chronic radiation proctitis	49	1	98.0	na	na
Takemoto	2012	Prospective	Hemorrhagic radiation proctopathy	12	2	83.3	na	na
Tam	2000	Retrospective	Proctitis	15	0	100.0	10.8 ± 2.57	13.3 ± 1.84
Tjandra	2001	Prospective	Hemorrhagic proctitis	12	6	50.0	11.18 ± 0.82	12.28 ± 0.55
Venkatesh	2002	Prospective	Radiation proctitis	40	1	97.5	na	na
Villavicencio	2002	Prospective	Hemorrhagic radiation proctopathy	21	0	100.0	na	na
Yeoh	2013	Prospective	Chronic radiation proctitis	17	1	94.1	14 (97–159)	13.6 (10.6–17.4)
Zinicola	2003	Retrospective	Radiation proctitis	12	2	83.3	na	na

**Table 3:** Study characteristics of selected studies on short-term and long-term complications after APC treatment.

Author	Proctitis	Total number of bleeding	Short-term complications	Number of perforations	Long-term complications	Rate of complications (%)
Alfadhli	Prospective	14	2 (vomiting, abdominal cramps, rectal pain, and fever)	0	0	14.30
Álvaro-Villegas	Chronic radiation proctitis	14	2 (rectal pain)	0	3 (rectal ulcers)	35.70
Ben	Hemorrhagic radiation proctitis	27	5 (anal or rectal pain, vagal symptoms, and colonic explosions without perforation in 2 and perforation in 1)	1	0	18.50
Canard	Radiation proctitis	30	6 (post treatment pain)	1	1 (extensive necrosis of the lower part of the rectum)	26.70
Chruscielewska-Kiliszek	Chronic radiation proctitis	62	1 (adynamic ileus)	0	35 (asymptomatic rectal ulcer in 30 and symptomatic rectal ulcers in 5)	58.10
Chutkan	Proctitis	12	0	0	0	0.00
de la Serna Higuera	Hemorrhagic radiation proctopathy	10	0	0	0	0.00
Dees	Chronic radiation proctitis	48	0	0	1 (recurrence of rectal bleeding)	2.00
Fantin	Proctitis	7	0	0	0	0.00
Hortelano E	Chronic radiation proctitis	30	1 (incontinence)	0	1 (rectal ulcer)	6.67
Kaassis	Proctitis	16	4 (transitory and minimal dysenteric)	0	1 (recurrence of rectal bleeding)	31.30
Karamanolis	Radiation proctitis	56	1 (colonic explosion without perforation)	0	2 (recurrence of rectal bleeding)	5.4
Latorre	Chronic radiation proctopathy	38	na	na	0	0.00
Lenz	Chronic radiation coloproctopathy	15	4 (anal pain in 2 cases and abdominal discomfort in 1, worsening of bleeding during treatment in 1)	0	2 (tapered feces without stenosis in 1, asymptomatic stenosis in 1)	40.00
Lpoez	Radiation proctopathy	19	0	0	1 (recurrence of rectal bleeding)	5.30
Onoyama	Chronic hemorrhagic radiation proctitis	24	0	0	0	0.00
Rolachon A	Proctitis and proctosigmoiditis	12	0	0	3 (chronic rectal ulcerations in 2 cases and rectal stenosis in 1 case)	25.00
Rotondano	Chronic radiation proctopathy	24	5 (mild bloating, cramping, anal pain)	0	1 (rectal stenosis)	25.00
Sait Dag	Radiation proctitis	21	4 (rectal pain and distension)	0	1 (recurrence of rectal bleeding)	23.80
Samy	Chronic proctitis	23	na	na	0	0.00
Sarah	Proctitis	11	0	0	7 (rectal stenosis in 2 patients, ulceration in 1, asymptomatic superficial ulceration in 4)	63.60
Sato	Hemorrhagic radiation proctopathy	65	8 (rectal pain)	0	4 (recurrence of rectal bleeding)	18.50
Sebastian	Radiation proctopathy	25	0	0	0	0.00
Silva	Proctosigmoiditis	28	3 (transient anal pain)	0	0	10.70
Smith	Proctitis	7	0	0	0	0.00
Swan	Chronic radiation proctitis	49	17 (proctalgia in 13 patients, rectal mucous discharge in 4, incontinence in 1, fever in 1, and bleeding in 1)	0	1 (rectal stricture)	36.00
Takemoto	Hemorrhagic radiation proctopathy	12	0	0	0	0.00
Tam	Proctitis	15	0	0	2 (rectal stricture)	13.30
Tjandra	Hemorrhagic proctitis	12	0	0	0	0.00
Venkatesh	Radiation proctitis	40	0	0	0	0.00
Villavicencio	Hemorrhagic radiation proctopathy	21	3 (rectal pain, tenesmus, and/or abdominal distention)	0	1 (recurrence of rectal bleeding)	19.00
Zinicola	Radiation proctitis	12	0	0	1 (rectosigmoid stenosis)	7.10

**Table 4:** Newcastle-Ottawa Scale for assessing quality of cohort studies.

Quality assessment scale	1	2	3	4	5	6	Total (max = 6)
Ben (2004)	∗	∗	∗	∗	∗	∗	6
Canard (2003)	∗	∗	∗	∗	∗	∗	6
Chruscielewska (2013)	∗	∗	∗	∗	∗	∗	6
Chutkan (1997)	∗	∗	∗	∗	∗	∗	6
Higuera (2006)	∗	∗	∗	-	∗	∗	5
Dees (1999)	∗	∗	∗	∗	∗	-	5
Fantin (2004)	∗	∗	∗	∗	∗	∗	6
Hortelano (2014)	∗	∗	∗	∗	∗	∗	6
Kaassis (2002)	∗	∗	∗	∗	∗	∗	6
Karamanolis (2009)	∗	∗	∗	∗	∗	∗	6
Latorre (2008)	∗	∗	∗	∗	∗	∗	6
Lpoez (2010)	∗	∗	∗	∗	∗	∗	6
Onoyama (2011)	∗	∗	∗	∗	∗	∗	6
Rolachon (2000)	∗	∗	∗	∗	∗	∗	6
Rotondano (2003)	∗	∗	∗	∗	∗	∗	6
Sait (2013)	∗	∗	∗	∗	∗	∗	6
Samy (2012)	∗	∗	∗	-	-	∗	4
Sarah (2001)	∗	∗	∗	∗	-	∗	5
Sato (2011)	∗	∗	∗	∗	∗	∗	6
Sebastian (2004)	∗	∗	∗	-	∗	∗	5
Silva (1999)	∗	∗	∗	∗	∗	∗	6
Smith (2001)	∗	∗	∗	∗	-	∗	5
Swan (2010)	∗	∗	∗	∗	∗	∗	6
Takemoto (2012)	∗	∗	∗	∗	∗	∗	6
Tam (2000)	∗	∗	∗	∗	∗	∗	6
Tjandra (2001)	∗	∗	∗	∗	∗	∗	6
Venkatesh (2002)	∗	∗	∗	-	-	∗	4
Villavicencio (2002)	∗	∗	∗	∗	∗	∗	6
Zinicola (2003)	∗	∗	∗	∗	∗	∗	6

1: representativeness of the exposed cohort; 2: ascertainment of APC exposure; 3: demonstration that outcome of interest was not present at start of study; 4: assessment of outcome; 5: was follow-up long enough for outcomes to occur; 6: adequacy of follow-up of cohorts. Asterisk (∗) indicated one score and hyphen (-) indicated zero score.
